# Altered Composition of Gut Microbiota in Depression: A Systematic Review

**DOI:** 10.3389/fpsyt.2020.00541

**Published:** 2020-06-10

**Authors:** Zahra Amirkhanzadeh Barandouzi, Angela R. Starkweather, Wendy A. Henderson, Adwoa Gyamfi, Xiaomei S. Cong

**Affiliations:** ^1^School of Nursing, University of Connecticut, Storrs, CT, United States; ^2^Center for Advancement in Managing Pain, School of Nursing, University of Connecticut, Storrs, CT, United States

**Keywords:** gastrointestinal microbiome, gut microbiome, microbiota, depression, major depressive disorder

## Abstract

Cumulative evidence shows a linkage between gut microbiota pattern and depression through the brain-gut microbiome axis. The aim of this systematic review was to identify the alterations of the gut microbiota patterns in people with depression compared to healthy controls. A comprehensive literature search of human studies, published between January 2000 and June 2019, was reviewed. The key words included gastrointestinal microbiome, gut microbiome, microbiota, depression, depressive symptoms, and depressive disorder. The systematic review adhered to the Preferred Reporting Items for Systematic Reviews and Meta-Analysis (PRISMA) guidelines. Nine articles met the eligibility criteria. Disparities in α-diversity and β-diversity of the microbiota existed in people with depression compared to healthy controls. At the phylum level, there were inconsistencies in the abundance of *Firmicutes*, *Bacteroidetes*, *and Proteobacteria*. However, high abundance in *Actinobacteria* and *Fusobacteria* phyla were observed in people with depression. On the family level, high abundance of *Actinomycineae*, *Coriobacterineae*, *Bifidobacteriaceae*, *Clostridiales incertae sedis XI*, *Porphyromonadaceae*, *Clostridiaceae*, *Lactobacillaceae*, *Streptococcaceae*, *Eubacteriaceae*, *Thermoanaerobacteriaceae*, *Fusobacteriaceae*, *Nocardiaceae*, *Streptomycetaceae*, *and* low abundance of *Veillonellaceae*, *Prevotellaceae*, *Bacteroidaceae*, *Sutterellaceae*, *Oscillospiraceae*, *Marniabilaceae*, and *Chitinophagaceae* were observed in people with depression. On the genus level, high abundance of *Oscillibacter*, *Blautia*, *Holdemania*, *Clostridium XIX*, *Anaerostipes*, *Anaerofilum*, *Streptococcus*, *Gelria*, *Turicibacter*, *Parabacteroides*, *Eggerthella*, *Klebsiella*, *Paraprevotella*, *Veillonella*, *Clostridium IV*, *Erysipelotrichaceae incertae sedis*, *Eubacterium*, *Parvimonas*, *Desulfovibrio*, *Parasutterella*, *Actinomyces*, *Asaccharobacter*, *Atopobium*, *Olsenella* and low abundance of *Coprococcus*, *Lactobacillus*, *Escherichia/Shigella*, *Clostridium XlVa*, *Dialister*, *Howardella*, *Pyramidobacter*, and *Sutterella* were found in people with depression. Alteration of gut microbiome patterns was evident in people with depression. Further evidence is warranted to allow for the translation of microbiome findings toward innovative clinical strategies that may improve treatment outcomes in people with depression.

## Introduction

Depression affects more than 300 million people of all ages globally (1), and is one of the leading causes of psychiatric disability (2). Outcomes of current depression psychotherapies are suboptimal with treatment failures common (3). Research shows that one in two patients does not sufficiently respond to depression treatment, 40% of responders relapse, and many non-responders deteriorate despite treatment (2, 4). Failure to enhance the treatment outcomes reflects the need for further research related to the pathophysiology of depression.

**Figure 1:**
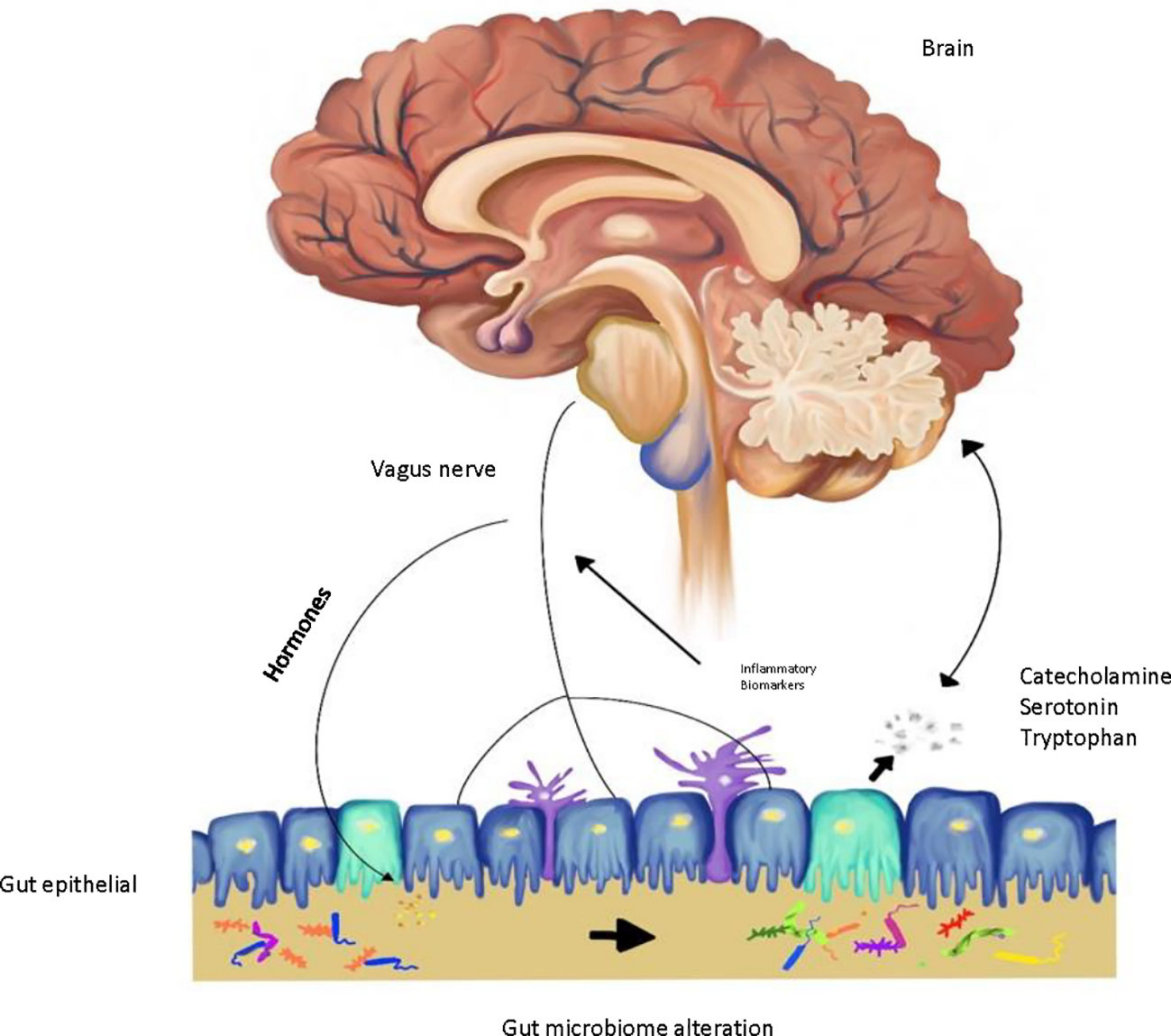
Gut-brain microbiome axis in depression.

Traditionally, the theories behind the pathophysiology of depression are attributed to neurotransmitters, stress hormones, neurotrophic factors, and circadian rhythms (5). The monoaminergic system plays an important role in depression with multiple interactions in the central nervous system (CNS) function (6). In recent studies, the gut microbiota has been introduced as a novel area of investigation of depression pathophysiology (2). The gut microbiota is composed of over 100 trillion microorganisms that affect host homeostasis (7–10). Gut microbes in the brain-gut axis communicate to the CNS *via* endocrine, nervous, and immune signaling mechanisms. The brain may affect the community pattern and function of the gut microbiota throughout the autonomic nervous system (ANS) through modulation of the intestinal transit *via* secretion, regional gut motility, and gut permeability (11) (**Figure 1**).

Numerous studies have investigated the underlying mechanisms of depression through the brain-gut microbiota axis. Changes in gut microbiota patterns result in immune activation through the bidirectional interactions between the gut and the brain, potentially yielding the generation of various types of psychiatric symptoms (12). Some animal studies implicate fecal “depression microbiota” transplantation modulates depression-like behavior in germ-free (GF) mice (13). Moreover, human studies demonstrated the lower abundance of *Lactobacillus* and *Bifidobacterium* in persons with depression (14). Anti-inflammatory effects of *Lactobacillus* and *Bifidobacterium* may explain the role of gut microbiota in this trajectory (14). Despite current theories that propose the role of gut microbiota in the pathophysiology of depression, the mechanism by which gut microbiota modulate depression-like behaviors remains controversial.

Differences in the microbiota community at various taxonomic levels have been reported in study participants living with depression compared to those without depression (15–18). The studies observed various gut microbiota compositions in persons with depression. However, the reported results are inconclusive and require definitive distinctions (18). The purpose of this systematic review is to summarize the primary results from recent human studies and provide evidence for future research in finding the patterns of the gut microbiota in people with depression.

## Materials and Methods

### Study Selection Criteria

The targeted study population for this review was people with depression, depressive symptoms, or major depression. The primary outcome measures were gut microbiome composition, including the α-diversity and β-diversity, and the abundance of bacteria at phylum, family, and genus taxonomic levels. The articles eligible for this review paper were original research articles, human research studies conducted in adults, written or available in English, and published between January 2000 and June 2019. All study designs from any country were included. Manuscripts were excluded if they were letters, book chapters, reviews, theses/dissertations, secondary data analyses, or case studies. Studies of people with depression and other chronic or comorbid disorders such as irritable bowel syndrome, inflammatory bowel disease (IBD), anorexia nervosa, and/or cancer were excluded.

### Information Sources and Search Strategies

The online databases of PubMed, CINAHL, Psych Info, and Scopus were searched for relations between depression and gut microbiome patterns with the terms gastrointestinal microbiome, gut microbiome, microbiota, gut flora, depression, depressive symptoms, depressive disorder, and major depressive disorder.

### Study Selection

The irrelevant articles, based on the titles, were excluded and the remainder were screened for relevance after review of the abstract and full-text independently by two researchers.

### Quality Control of Selected Studies

The methodological quality of the included studies was evaluated with a modified Newcastle Ottawa scale for cross-sectional studies (19). This scale was used to assess the quality of cross-sectional studies. The assessed criteria include selection, comparability, and outcomes. The maximum score that represents the highest possible quality is 10.

### Data Analysis

The findings of gut microbiome patterns in persons with depression were categorized based on the bacteria diversity and taxonomic levels. Thus, the gut microbiome was grouped based on α-diversity (the richness and evenness of the microbial community), β-diversity (the compositional dissimilarity among the microbiome community), the abundance and proportion of bacteria at phylum, family, and genus levels.

## Results

### Selection of Literature

The initial online database search produced 616 articles, of which 98 were duplicates, and 440 excluded with no related outcome. The full-text articles assessed for eligibility were 78, of which 69 were ineligible because of comorbidity and nine full-text were eligible for the systematic review (**Figure S1**).

### Characteristics of the Samples and Methodologies Used

The subjects of the studies were adults with major depressive disorder and healthy controls aged ≥18 years old. Depression was diagnosed clinically using Mini-International Neuropsychiatric Interview (MINI), Diagnostic and Statistical Manual of Mental Disorders (DSM-IV), Diagnostic and Statistical Manual of Mental Disorders Text Revision (DSM-IV-TR), and International Classification of Diseases (ICD-10). Depression severity was assessed with a variety of standardly accepted instruments including Hamilton Depression Rating Scale (HDRS), Montgomery–Åsberg Depression Rating Scale (MADRS), or Beck Depression Inventory. The selected studies compared the fecal gut microbiota composition between people with depression and healthy controls. Six studies were conducted in China, one in Norway, one in Ireland, and one in Japan. The study design for eight studies was cross-sectional, and one was a partially blinded observational study.

The microbiome analysis involved sequencing of hypervariable regions V1–V5 of the 16S rRNA genes. Different primers were used in the studies including bar-coded universal primers containing linker sequences for 454-pyrosequencing, 27F and 533R primers. Techniques including polymerase chain reaction (PCR), reverse transcription-quantitative PCR (RT-qPCR), pyrosequencing, Illumina sequencing, and Illumina sequencing MiSeq platform were utilized for DNA sequencing. Specifically, comparative meta-proteomics analysis by phylogenetic analysis of the bacterial peptides was utilized in one study (20). Sequential clustering of operational taxonomic units (OTUs) was identified at 95–100% nucleotide similarity to calculate the relative abundances of microbiota at different Levels (15–17, 21–25). More than 80% of the articles were published within the years 2016 to 2018.

### Alteration of the Gut Microbiome Community in Depression

#### Altered Gut Microbiota Diversity and Richness in Depression

Microbial α-diversity was reported in six studies by using different indices and methods, such as Shannon index, Simpson index, phylogenetic diversity, total observed species and Chao 1 (26). Three studies reported contradictory results regarding α-diversity within bacterial community based on the Shannon index. Jiang et al. observed higher α-diversity, whereas Liu et al. reported lower α-diversity and Kelly et al. indicated no significant difference in this index (15, 16, 23). In terms of measuring α-diversity using other methods, Naseribafrouei et al. found no significant differences between people with depression and healthy controls with respect to Simpson’s index (25). Kelly et al. reported a decrease in total observed species and Chao 1 richness in people with depression (16). Zheng et al. and Chen et al. indicated no significant difference between people with depression and healthy people with respect to phylogenetic diversity (22, 24). However, Kelly et al. reported a decrease in phylogenetic diversity in people with depression (16).

Gut microbial β-diversity, measured using the Bray-Curtis Dissimilarity Index, was reported in three studies. Kelly et al. and Zheng et al. reported significant differences in β-diversity between people with depression and healthy controls. However, Jiang et al. did not obtain an estimate of this index due to a significant inter-individual variability (15, 16, 24). Therefore, no consistent directional alteration of the microbial diversity was found in people with depression compared to healthy controls.

#### Altered Gut Microbiota Composition in Depression

On the phylum level, five phyla including *Firmicutes*, *Bacteroidetes*, *Actinobacteria*, *Proteobacteria*, and *Fusobacteria* were reported in the seven studies with contradictory results in their abundance between people with depression and healthy control groups. Chen et al. and Lin et al. reported higher abundance of *Firmicutes*, whereas, Jiang et al. and Liu et al. found lower abundance of this phylum (15, 17, 22, 23). Interestingly, Zheng et al. reported no difference of *Firmicutes* phylum between people with depression and healthy controls (24). *Bacteroidetes* as another phylum was mostly reported in the studies. Jiang et al. and Liu et al. reported high abundance of this phylum, whereas Naseribafrouei et al., Zheng et al., Lin et al., Chen et al., and Chen et al. found lower abundance of this phylum in people with depression (15, 20, 22, 24, 25). Higher abundance of *Actinobacteria* were reported by Jiang et al., Zheng et al., Chen et al., and Chen et al. (15, 20, 22, 24) in people with depression. While, Jiang et al. reported high abundance of *Proteobacteria*, Chen et al. found lower abundance of this phylum in people with depression. Also, Jiang et al. reported high abundance of *Fusobacteria* in people with depression.

On the family level, studies reported higher abundance of 13 families including *Actinomycineae, Coriobacterineae, Bifidobacteriaceae, Clostridiales incertae sedis XI, Porphyromonadaceae, Clostridiaceae, Lactobacillaceae, Streptococcaceae*, *Eubacteriaceae, Thermoanaerobacteriaceae, Fusobacteriaceae, Nocardiaceae*, and *Streptomycetaceae* and lower abundance of seven families including *Veillonellaceae, Prevotellaceae, Bacteroidaceae, Sutterellaceae, Oscillospiraceae, Marniabilaceae*, and *Chitinophagaceae* in people with depression (15, 16, 20, 22, 24). Contradictory results were found on the abundance of six families, namely, *Lachnospiraceae, Ruminococcaceae, Acidaminococcaceae, Enterobacteriaceae, Erysipelotrichaceae*, and *Rikenellacea* in people with depression (15, 16, 20, 22, 24, 25). On the genus level, studies reported high abundance of 26 genera including *Oscillibacter, Blautia, Holdemania, Clostridium XIX, Anaerostipes, Lachnospiracea incertae sedis, Anaerofilum, Streptococcus, Gelria, Turicibacter, Parabacteroides, Eggerthella, Klebsiella, Streptococcus*, *Paraprevotella, Veillonella, Clostridium IV, Erysipelotrichaceae incertae sedis, Eubacterium, Parvimonas, Desulfovibrio, Parasutterella, Actinomyces, Asaccharobacter, Atopobium*, and *Olsenella* and a low abundance of eight genera including *Coprococcus*, *Lactobacillus*, *Escherichia/Shigella*, *Clostridium XlVa*, *Dialister*, *Howardella*, *Pyramidobacter*, and *Sutterella* in people with depression (15, 16, 22–24). However, a lack of congruence existed across investigations for the abundance of *Bifidobacterium, Roseburia, Lachnospiracea incertaesedis, Megamonas, Clostridium XI, Bacteroides, Prevotella, Alistipes, Phascolarctobacterium, Faecalibacterium*, and *Ruminococcus* (15, 17, 23, 24) (**Tables 1** and **2**).

**Table 1:** Studies on gut microbiome composition in depression.

Authors/Year Country	Study Design	Study Subjects	Biospecimen	Diagnosis/Severity of Depression Scales	Mean severity of depression at baseline	Microbiological Analysis	Total score of quality assessment (0–10)
Jiang et al. (15) China	Cross-sectional	n = 29 patients with depression [Active-Major Depressive Disorder, (A-MDD)] n = 17 patients during response to antidepressant treatment [Responded-Major Depressive Disorder (R-MDD)] n = 30 healthy controls Age: 18–40 years	1) Fecal samples 2) Blood samples (Serum tumor necrosis factor-a (TNF-a), interleukin-1b (IL)-1b, IL-6, and brain-derived neurotrophic factor (BDNF) levels	Diagnostic and Statistical Manual of Mental Disorders Fourth Edition (DSM IV), Hamilton’s Depression Scale (HAMDS), Montgomery–Asberg Depression Rating Scale (MADRS)	HAMDS: 29.8 MADRS: 27.4	16S rRNA genes (V1–V3 regions), PCR, Pyrosequencing. α-diversity, ß-diversity, bacterial taxonomic composition	7
Kelly et al. (16) Ireland	Cross-sectional	n = 34 patients with depression n = 33 healthy controls Mean age: 45.8	Fecal sample Salivary cortisol Plasma Tryptophan and kynurenine pathway metabolites Plasma levels of IL-6, IL-8, TNF-a, and CRP Lipopolysaccharide binding protein	Diagnostic and Statistical Manual of Mental Disorders (DSM IV)/Hamilton rating scale for Depression (HAMD 17)/Beck Depression Inventory	HAMD: 19.5 Beck Depression: 32.4	16S rRNA genes, illumina MiSeq platform. α-diversity, ß-diversity, bacterial taxonomic composition	6
Aizawa et al. (21) Japan	Cross-sectional	n = 43 patients with depression n = 57 healthy controls Mean age: 40 years	Fecal samples	Diagnostic and Statistical Manual of Mental Disorders, 4th ed. (DSM-IV), Hamilton depression rating scale	HAM-D: 16.9	16S rRNA genes, Reverse Transcription quantitative PCR, bacterial taxonomic composition	5
Lin et al. (17) China	Cross-sectional	n = 60 patients with depression n = 60 healthy controls Age: 20–85 years	Fecal samples	Diagnostic and Statistical Manual of Mental Disorders (DSM-IVTR), Hamilton depression rating scale (HAM-D) scale	HAM-D ≥23	16S rRNA genes (V3-V4), Illumina MiSeq platform. bacterial taxonomic composition	6
Naseribafrouei et al. (25) Norway	Partially blinded observational study	n = 37, patients with depression n = 18 healthy controls Mean age: 48 years	Fecal samples	ICD-10, Montgomery-Asberg Depression Rating Scale (MADRS)	MADRS: 26.3	16S rRNA genes, PCR, Illumina sequencing. α-diversity, ß-diversity, bacterial taxonomic composition	5
Chen et al. (20) China	Cross-sectional	n = 10 people with depression n = 10 healthy controls Age:18–65 years	Fecal samples	Diagnostic and Statistical Manual of Mental Disorders, 4th ed. (DSM-IV), Hamilton’s Depression Scale	HAMDS: 25.6	Protein, Liquid chromatography-tandem mass spectrometry	6
Liu et al. (23) China	Cross-sectional	n = 40 IBS-D patients n = 15 people with depression n = 25 comorbidity patients with both IBS and depression n = 20 healthy controls Age >18 years	Fecal samples	Diagnostic and Statistical Manual of Mental Disorders, 4th edition (DSM-IV)/Self-Rating Depression Scale (SDS)	SDS:60	16S rRNA genes (V1 to V3 region), PCR, Pyro sequencing. α-diversity, ß-diversity, Bacterial taxonomic composition	7
Zheng et al. (24) China	Cross-sectional	n = 58 patients with depression, n = 63 healthy controls; age >18 years	Fecal samples	DSM-IV-TR, Hamilton Depression Rating Scale	HDRS: 22.8	16S rRNA genes (V3-V5 regions), PCR, illumina sequencing. α-diversity, ß-diversity, bacterial taxonomic composition	7
Chen et al. (22) China	Cross-sectional	n = 24 people with depression, n = 24 healthy controls; age >18 years old (18–60)	Fecal samples	Diagnostic and Statistical Manual of Mental Disorders IV, Hamilton Depression Rating Scale (HDRS-17)	HDRS: 23.04	16S rRNA genes (V3–V5 regions), PCR, Pyro sequencing. α-diversity, ß-diversity, Bacterial taxonomic composition	6

**Table 2:** Fecal bacteria diversity and abundance in people with depression.

Alpha diversity	Beta diversity	Phylum	Family	Genus
Shannon index: Jiang ↑ Liu ↓ Kelly ↔	Jiang ↔ Kelly * Zheng *	*Firmicutes* Jiang ↓ Zheng ↔ Lin ↑ Liu ↓ Zhi Chen ↑	*Acidaminococcaceae* Jiang ↑, Zheng ↓	*Phascolarctobacterium* Jiang ↑, Zheng ↓
			E*rysipelotrichaceae* Jiang ↓, Zhi Chen ↑	*Holdemania* Kelly ↑
Simpson index: Naseribafrouei ↔ Total observed species: Kelly ↓			*Lachnospiraceae* Jiang ↓, Naserabadi ↓, Zheng ↓, Zhi Chen ↑, Chen ↑	*Clostridium XIX* Jiang ↑ (A-MDD) *Anaerostipes* Zheng ↑, Chen ↑ *Lachnospiracea incertae sedis* Jiang ↑ (A-MDD) *Roseburia* Jiang ↑, Zheng ↓, Chen ↑
Chao 1: Kelly ↓ Phylogenetic diversity: Zheng ↔, Chen ↔, Kelly ↓			*Ruminococcaceae* Jiang ↓, Zheng ↑, Zhi Chen ↑, Chen ↑	*Faecalibacterium* Jiang ↓, Zheng ↓, Zhi Chen ↓, Chen ↑ *Ruminococcus* Jiang ↓, Zheng ↑ *Anaerofilum* Kelly↑
			*Veillonellaceae* Jiang ↓, Zheng ↓	
			*Lactobacillaceae* Zheng ↑	*Lactobacillus* Aizawa ↓ *Blautia* Jiang ↑, Zheng ↑, Chen ↑ *Clostridium XIX* Jiang ↑ *Lachnospiracea incertae sedis* Jiang ↑, Liu ↓ *Coprococcus* Zheng ↓, Liu ↓ *Clostridium XlVa* Zheng ↓, Liu ↓
			*Streptococcaceae* Zheng ↑	*Streptococcus* Lin ↑
			*Eubacteriaceae* Zheng ↑	
			*Erysipelotrichaceae incertae sedis* Zheng ↑	
			*Thermoanaerobacteriaceae* Kelly ↑	*Gelria* Kelly ↑
			*Selenomonadaceae*	*Megamonas* Jiang ↑, Zheng ↓
			O*scillospiraceae* Zhi Chen ↓	*Oscillibacter* Jiang ↑ (A-MDD), Naserabadi ↑
			*Dialisteraceae*	*Dialister* Jiang ↓, Kelly ↓
			*Clostridiaceae* Zhi Chen ↑	*Clostridium XI* Lin ↑, Liu ↓
			*Turicibacteraceae*	*Turicibacter* Kelly ↑
		*Bacteroidetes* Jiang ↑, Liu↑, Naserabadi ↓, Zheng ↓, Lin ↓, Chen ↓, Zhi Chen ↓	*Porphyromonadaceae* Jiang ↑, Zhi Chen ↑	
			*Rikenellaceae* Jiang ↑, Zheng ↓, Zhi Chen ↓	*Alistipes* Jiang ↑, Naserabadi ↑, Zheng ↓
			*Bacteroidaceae* Jiang ↓ (A-MDD), Zheng ↓	*Bacteroides* Jiang ↓ (A-MDD), Chen ↑, Liu↑ *Prevotella* Jiang ↓, Kelly ↓, Liu↑, Lin↑ *Paraprevotella* Liu↑, Kelly ↑
			*Chitinophagacea* *Marniabilaceae* Zhi Chen ↓ *Prevotellaceae* Jiang ↓, Kelly ↓, Zhi Chen ↓	
			*Clostridiales incertae sedis XI* Zheng ↑	
				*Lactobacillus* Aizawa ↓
			*Tannerellaceae*	*Parabacteroides* Jiang ↑
		*Actinobacteria* Jiang ↑, Chen ↑, Zheng ↑, Zhi Chen ↑	*Actinomycineae* Zheng ↑, Zhi Chen ↑	
			*Coriobacterineae* Zheng ↑, Chen ↑	
			*Bifidobacteriaceae* Zhi Chen ↑	*Bifidobacterium* Aizawa ↓, Chen ↑
			Eggerthellaceae Nocardiaceae Zhi Chen ↑ Streptomycetaceae Zhi Chen ↑	*Eggerthella* Kelly ↑, Chen ↑
		*Proteobacteria* Jiang ↑, Zhi Chen ↓	*Enterobacteriaceae* Jiang ↑, Zhi Chen ↓	*Escherichia/Shigella* Jiang ↓ (R-MDD) *Klebsiella* Lin↑
			*Sutterellaceae* Zheng ↓, Zhi Chen ↓	
			*Burkholderiaceae*	*Parasutterella* Jiang ↑ (A-MDD)
		*Fusobacteria* Jiang ↓ (R-MDD), ↑ (A-MDD)	*Fusobacteriaceae* Jiang ↑ (A-MDD)	

↑, high abundance in people with depression; ↓, low abundance in people with depression; ↔, direction not deciphered; *, significant difference; A-MDD, active-MDD; R-MDD, recovering-MDD.

#### Altered Gut Microbiota Composition Association With Antidepressant Medications

Some studies addressed the effects of antidepressant medications on microbiome composition. Jiang et al. observed a high abundance of *Bacteroidetes* and *Proteobacteria* and a low abundance of *Firmicutes*, *Actinobacteria*, and *Fusobacteria* phyla in response to antidepressant treatment with selective serotonin reuptake inhibitors (SSRIs) or serotonin–norepinephrine reuptake inhibitors (SNRIs) (15). On the family level, higher abundance of *Bacteroidaceae*, *Acidaminococcaceae*, *Porphyromonadaceae*, *Enterobacteriaceae*, and *Rikenellaceae* were reported in response to anti-depressant medications in people with depression compared to healthy control group. Additionally, five genera, including *Alistipes*, *Bacteroides*, *Parabacteroides*, *Phascolarctobacterium*, and *Roseburia*, increased in in people with depression after antidepressant treatment (15). Lin et al. (17) evaluation of the bacterial composition at the phylum level in people with depression under treatment of escitalopram (SSRIs) across three visits for one month showed no differences in the microbial community during the three different visits. Also, Zheng et al. reported no significant correlation between antidepressant treatment and bacterial composition, although the majority of people with depression were drug-naïve and the remaining participants used SSRIs, SNRIs, or tricyclic antidepressants (TCAs) (24). Similarly, Aizawa et al. observed no significant correlation between anti-depressant medication (Imipramine) dosage and bacterial counts (21).

## Discussion

The systematic review revealed some differences in the gut microbiota diversity, the richness and evenness of microbes were different in people living with depression as compared to healthy adults. No consensus in the α-diversity and β-diversity was evident. Additionally, different quantities of bacterial abundance were present at the family and genus levels. There was no consensus in abundance of *Firmicutes* even though most studies reported bacteria at family and genus levels belonged to the *Firmicutes* phylum among people with depression. Moreover, decrease in *Bacteriodetes* and increase in *Actinobacteria* abundance were observed in people with depression in the reviewed studies. The most common bacteria of interest were *Firmicutes*, followed closely at both family and genus level by *Bacteriodetes* and *Actinobacteria* phyla.

The findings from the systematic review illustrated inconsistencies in in the abundance of *Firmicutes*, a lower abundance of *Bacteroidetes* and a higher abundance of *Actinobacteria* phyla among people with depression. *Firmicutes* and *Bacteriodetes*, as the two major phyla in fecal microbial flora showed altered abundance and correlated to inflammatory conditions such as IBDs (17). A low abundance of *Bacteroidetes* has also been associated with obesity (25). Recently, research shows that colonization of GF mice from human depressed microbiota characterized by alterations in *Firmicutes*, *Actinobacteria*, and *Bacteroidetes* induced depression-like behavior in mice (24).

There was inconsistency among the abundance of bacteria at different taxonomic levels. High abundance of *Oscillibacter*, *Parabacteroide*, *Klebsiella*, *Paraprevotella*, *Veillonella*, *Desulfovibrio*, *Parasutterella*, and *Paraprevotella* as a gram-negative bacteria in people with depression may explain the contribution of microbiota in development/maintenance of depression. Gram-negative bacteria contain lipopolysaccharides (LPS) in the leaflet of the outer cell membrane (27). Studies show that LPS interacts with macrophages and stimulates the immune response through the release of pro-inflammatory cytokines (27). Some studies support this mechanism by reporting increased levels of proinflammatory cytokines interleukin, including IL-1β and IL-6, and decreased levels of anti-inflammatory cytokines, including IL-4 and IL-10 in people living with depression (28, 29). Thus, the gut microbiota alterations may modulate the inflammatory response in people with depression by attenuation of pro-inflammatory cytokines (30–33). Research indicate that the administration of probiotics such as *Lactobacillus rhamnosus* modulates the immune system through the prevention of the induction of IL-8 by TNF-α in human colon epithelial cell lines and modulates inflammation through the generation of regulatory T cells (34). Moreover, other studies suggest an anti-inflammatory effect of *Bifidobacterium*, *Faecalibacterium*, and *Lactobacillus* on stress responses and depressive disorders (15, 21, 35). Thus, underrepresentation of these genera in people with depression may verify elevated levels of inflammatory biomarkers in depressed patients. Current literature link microbiome dysbiosis with depression and related biomarkers, although the patterns of gut microbiome still requires definitive distinctions.

Traditionally, alteration of neurotransmitters may lead to depression (36). The gut microbiota are able to influence the body neurotransmitters levels by stimulate the CNS and the gut *via* the production of metabolites (32, 37). The produced metabolites may affect the body’s neurotransmitters and eventually lead to depression. For instance, short-chain fatty acids (SCFAs) can stimulate the release of serotonin in the gut (38). Also, Microbial metabolites may affect the central neurotransmitters by activating afferent nerve fibers (34). Thus, produced metabolites by gut microbiota may affect emotional behavior by influencing the body’s neurotransmitters either directly or indirectly.

The vast evidence from animal studies supports the hypothesis that gut microbiota plays a key role in CNS function, mainly through inflammation and the hypothalamic-pituitary-adrenal (HPA) axis (15), although the pathways linkage between the gut bacteria and the brain are not entirely understood, stress-induced altered intestinal permeability may play a role. Specifically, gut microbiota alterations and increased translocation of bacterial endotoxins due to a compromised gut barrier is linked to activation of the immune system and HPA axis (15). While a causal relationship is yet to be fully established, bacterial translocation products may lead to an increased production of inflammatory biomarkers (15, 35, 39).

Multiple potential drivers contributed to microbiome alteration in people with depression. These drivers may impact the bidirectional communication between the CNS and gut microbiota which eventually lead to depressive-like behavior (40). A few of drivers include environment, genetics, mode of birth and early exposure to stress, may play roles as potential drivers for alteration of gut microbiota pattern and differential signaling in the gut-brain axis (41, 42).

The other important factor is pattern of bacteria. It is important that the combination of the different taxa might enhance the pathogenic effect (27). The combination effects can be proved in animal studies. For instance, combination of *E.coli* and *B. fragilis* was needed for development of abscess in rats. Neither *E.coli* and *B. fragilis* alone cannot provoke abscess formation (43, 44). Thus, presence of various gram-negative bacteria at the same time in fecal microbiota of people with depression may be an indicator of depression.

The studies included herein were conducted in various geographic locations. Cultural and lifestyle differences may be factors that influence the pattern of the gut microbiota. Research shows that dietary differences between Eastern and Western countries may impact the bacterial composition and diversity (15). For instance, a high-fat diet may increase the abundance of the gram-negative bacteria in the gut which may lead to an increase in LPS concentration and stimulation of the immune system (27). Therefore, attention to the dietary pattern may also help to clarify the role of the gut microbiome in development/maintenance of depression.

Most of the included studies did not analyze the relationship between diet and gut microbial composition. Further studies are required to assess the role of dietary intake on gut microbiota pattern.

The use of diverse anti-depressant medications was observed to impose a variety of alterations in the bacterial community, which made it difficult to predict the bacterial community pattern in medicated depressed patients. Some animal studies have demonstrated both increases and decreases in the relative abundance of major phyla, notably, *Firmicutes* and *Bacteroidetes* during chronic antipsychotic medication administration (45). Hence, it remains unclear whether the gut pattern alternations are caused by depressive symptoms or anti-depressant medications.

### Limitations of the Review

Results from the studies synthesized in this review indicated inconsistent results regarding gut microbiome patterns and diversity in people with depression compared to healthy controls. The variations in findings may be attributed to the difference in methodology and population of the included studies. Most of the studies used 16S rRNA sequencing; however, one study utilized phylogenetic analysis of bacterial peptides which influence comparability of the studies. The analytical methods were different across studies involving various regions (V1–V5), various cut off points for clustering OTUs and using different scales for diagnosis of depression which may affect the results. Difference in studies’ population, including age, diet, weight, the geographic location, host genetics, and behavioral factors may influence the pattern of the gut microbiome. Ultimately, the findings consistently demonstrated that depression is associated with marked alterations in gut microbiota composition, although the specific alterations still require further study.

## Conclusion

The area of research on the role of microbiota in the brain-gut axis in development or maintenance of depression is still limited. The results of the included studies showed that the characteristics of gut microbiota in people with depression compared to healthy adults are inconsistent. There are conflicting reports on microbial diversity as well as the abundance of bacteria at phyla, family, and genus taxonomic levels in people with depression. The inconsistency suggests that there may be confounding factors within these complicated relationships. Furthermore, differences among taxonomic levels suggest that increased bacterial translocation and intestinal permeability may play a role in the pathophysiology of depression. Nonetheless, further studies are strongly suggested.

A higher bacterial diversity is potentially beneficial to human health, although the role in the gut-brain axis remains subjective to debate. The precise consequences of difference in bacterial diversity for people with depression thus remain unclear. The reported differences in diversity and composition of gut bacteria in this review paper are influenced by population characteristics including age, diet, health status, antibiotic treatment, as well as geographic location (15). The potential contributions of the demographic factors may highlight the role of psychological and social factors in the development of depression. The changes in the microbial patterns of fecal samples deserve more attention using a comprehensive approach in the future. Understanding the pattern of gut microbiota pattern may lead to novel strategies such as appropriate pre/probiotics in treatment of people with depression. Depression as a comorbid condition is among top 10 symptoms that is reported in chronic diseases such as cancer (46). Shedding light on the pattern of gut microbiota in depression may provide better insight in comorbidity between depression and other chronic conditions.

## Author Contributions

ZB, XC, WH, AG, and AS contributed to writing and editing the manuscript. The authors agree to be accountable for the content of the work.

## Funding

Funding was provided to the University of Connecticut, School of Nursing. 1P20NR016605-01 (PI: AS; pilot PI: XC), and RO1 NR016928 (PI: XC) and American Nurses Foundation (PI: ZB).

## Conflict of Interest

The authors declare that the research was conducted in the absence of any commercial or financial relationships that could be construed as a potential conflict of interest.
