# Supplementary figures and images for: Altered Composition of Gut Microbiota in Depression: A Systematic Review
Source: Front Psychiatry. 2020 Jun 10;11:541. doi: 10.3389/fpsyt.2020.00541 (PMC7299157; doi:10.3389/fpsyt.2020.00541)

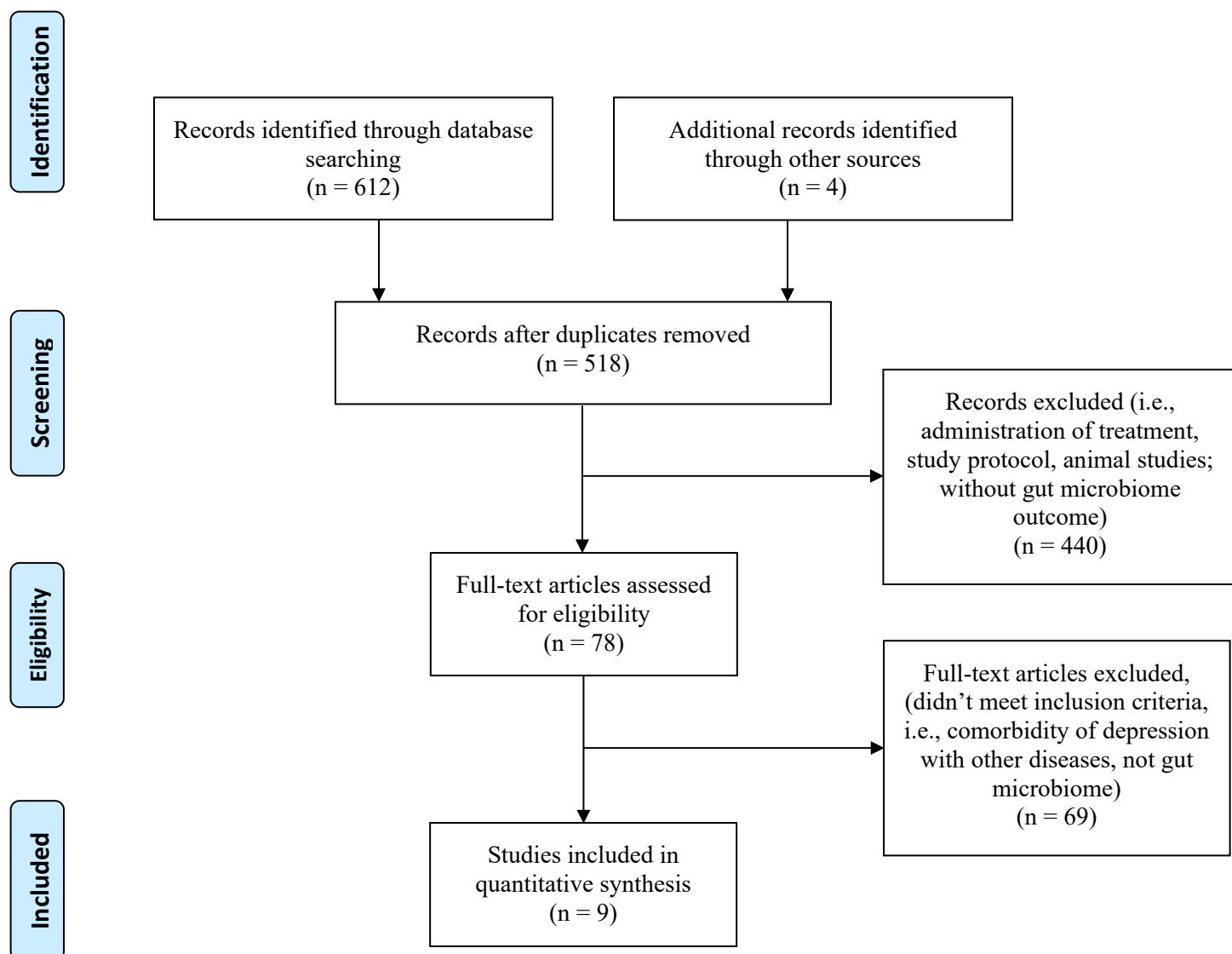

Figure S1: PRISMA flow diagram of the studies of gut microbiome pattern in depression.

Supplement: Supplementary file 1 [file Image_1.pdf]
